# Thoracic Block Technique Associated with Positive End-Expiratory Pressure in Reversing Atelectasis

**DOI:** 10.1155/2015/490326

**Published:** 2015-03-26

**Authors:** Luciana Carnevalli Pereira, Ana Paula de Souza Netto, Fernanda Cordeiro da Silva, Silvana Alves Pereira, Cristiane Aparecida Moran

**Affiliations:** ^1^University Nove de Julho (UNINOVE), São Paulo, Brazil; ^2^Ana Bezerra University Hospital (HUAB/EBSERH) and Federal University of Rio Grande do Norte (UFRN/FACISA), RN, Brazil

## Abstract

A preschool four-year-old male patient had been admitted to the Mandaqui Hospital with a diagnosis of lobar pneumonia, pleural effusion, and right lung atelectasis. Treatment consisted of antibiotics and physiotherapy sessions, using a technique described in the literature as Insufflation Technique to Reverse Atelectasis (ITRA), which consists of a thoracic block of healthy lung tissue, leaving only the atelectasis area free, associated with the use of invasive or noninvasive mechanical ventilation with positive airway pressure for reversal of atelectasis. Two physiotherapy sessions were conducted daily. The sessions lasted 20 minutes and were fractionated into four series of five minutes each. Each series bilateral thoracic block was performed for 20 seconds with a pause lasting for the same time. Associated with the thoracic block, a continuous positive airways pressure was used using a facial mask and 7 cm H_2_O PEEP provided via CPAP. *Conclusion*. ITRA technique was effective in reversing atelectasis in this patient.

## 1. Introduction

Despite the advances of mankind in recent years, high rates of infant mortality are still verified mainly in developing countries. Among the five main causes are the acute respiratory infections, responsible for the death of about three million children under five years per year [1, 2].

Among the acute respiratory infections, the community-acquired pneumonia represents the most severe form of acute respiratory infections, with an annual incidence of 150 million new cases, of which more than 11 million cases require hospitalization [2, 3].

In Brazil, about 48% of children with pneumonia are aged between one and four years; in these cases the bacterial causes gain more importance and are associated with increased risk of complications; family socioeconomic factors and child nutritional status also are involved. The main complications are pleural effusions and atelectasis, these being the main determinants of clinical worsening [2, 4, 5].

About 50% to 70% of pleural effusions in children are caused by infections secondary to pneumonia, which are discovered due to a chest X-ray for initial diagnosis and reversed with the same antibiotic treatment for community-acquired pneumonia. However, when there is no response to treatment, pleural effusion may have increased its volume and evolve into the so-called complicated pleural effusions and atelectasis [6, 7].

Atelectases are pulmonary changes due to bronchial obstruction caused by inflammation and are associated with functional consequences such as changes in oxygenation, decreased lung compliance, increased pulmonary vascular resistance, overexpansion of adjacent alveolar units, and lung injury. After the collapse of a segment or pulmonary lobe, the alveoli ventilation decreases, while the slightly decreased perfusion results in an area with low ventilation/perfusion (V/Q), causing consequent morbidities [8].

Scientific literature describes a technique known as ITRA, which is used to reverse atelectasis, Insufflation Technique to Reverse Atelectasis. The intervention consists in taking all the healthy lung tissue to an exhalation position and retaining them through thoracic block, leaving only the atelectasis area free, associated with the use of invasive or noninvasive mechanical ventilation with continuous positive airway pressure (CPAP). This positive pressure produces increased oxygenation, as it promotes the reexpansion of previously collapsed areas, decreases intrapulmonary pressure and increases the gas exchange surfaces, promotes the V/Q relation improvement, and decreases the work of breathing [8–10].

Although ITRA encourages the treatment of atelectasis in different ages [8–10], new studies that provide a better assessment of their effectiveness and a better prognosis in children shall be encouraged, because the pulmonary complications caused by community-acquired pneumonia are an important cause of morbidity in children aged zero to five years. This study aims to evaluate the efficiency of ITRA in reversing atelectasis.

## 2. Case Report

This case report was approved by the Committee of Research Ethics at University Nove de Julho, under the protocol number 40325.

**Figure 1:**
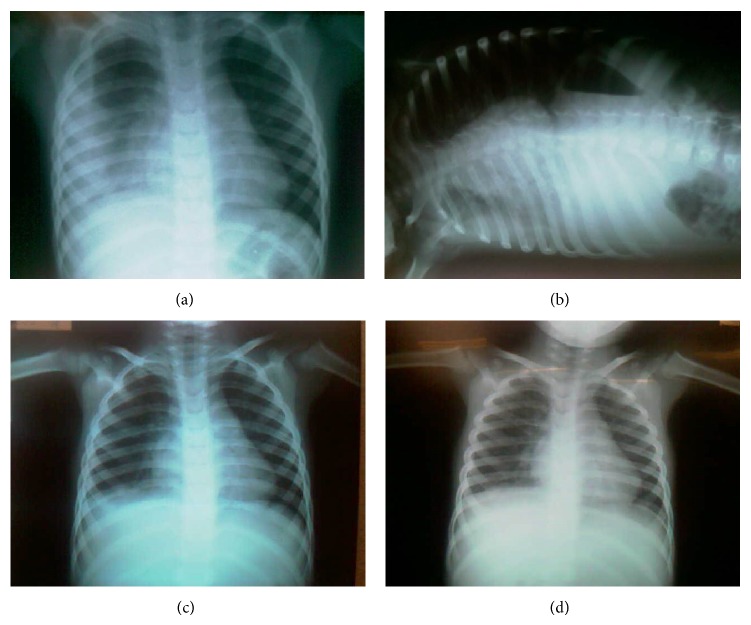
(a) Chest radiograph on the anteroposterior incidence, on day 1 of hospitalization and 15th day of clinical symptoms of pneumonia, showing the lobar pneumonia and a beginning of atelectasis in the right hemithorax. (b) Chest radiography on the incidence of Laurell on 1st day of admission showing pleural effusion in the right hemithorax. (c) Chest radiography on the posterior-anterior incidence on the 4th day of hospitalization, showing atelectasis improvement. (d) Chest radiography on the posterior-anterior incidence on the last day of hospitalization showing atelectasis reversal.

A 4-year-old child, residing in the São Paulo city, proceeding from Medical Ambulatory Care, with a medical history of dry cough initiated on August 5, 2012. The patient had been taken to the Primary Health Care Unit for three times, received antiallergic, and presented two fever's episodes (38°C and 38.5°C) and a vomit episode after cough. The patient was referred to the Mandaqui Hospital on August 20, 2012, and remained hospitalized with clinical and radiographic diagnosis of right lobar pneumonia with opacification of two-thirds of the right hemithorax and pleural thickening, being initiated on an antibiotic therapy with Crystalline Penicillin (200.000 U/kg/day), four times a day for 48 hours (Figures 1(a) and 1(b)).

During the assessment performed by the team of child surgery on August 21, a conservative treatment without the need for surgical intervention (thoracic drainage) was opted for. The ultrasonography performed on August 22, 2012, presented pleural effusion with septa in between and volume estimated at approximately 100 mL. After the pleural effusion diagnosis the patient underwent new antibiotic therapy, Ceftriaxone 600 mg two times a day (70 mg/kg/day) for 10 days and respiratory physiotherapy. Two daily sessions were performed during the period of hospitalization, except on weekends when there was no physiotherapist in attendance. During the 20-minute sessions, the ITRA was performed which consists of a thoracic block technique associated with the continuous positive airways pressure (CPAP), using a facial mask and 7 cmH_2_O PEEP provided via CPAP [11, 12]. This 20-minute sequence was fractionated in four series of five minutes each. During each series bilateral thoracic block was performed for 20 seconds with a pause lasting for the same time, and the healthy lung airflow was reduced by thoracic block [11]. The heart and respiratory rates were checked as well as the patient's O_2_ saturation before and immediately after the application of the technique and 10 minutes after the end of the session.  Table 1 presents the average behavior of clinical variables assessed.

After four days of the beginning of the sessions, a new radiography was performed and showed an image improvement (Figure 1(c)), remaining without complications until September 5, 2012 (Figure 1(d)), when the patient was discharged.

## 3. Discussion

The research demonstrated the reversal of atelectasis after thoracic-abdominal block's association with the CPAP technique known as ITRA, after 20 physiotherapy sessions. Despite the innovative name for Brazilian professionals, it consists of procedures routinely used by physiotherapists in hospitals in various clinical conditions [8–10].

The intervention of respiratory physiotherapy is still questioned regarding its benefits and efficiency in the treatment of respiratory disorders. Studies conducted using conventional physiotherapy techniques (tapping, percussion, postural drainage, and oropharyngeal aspiration assisted cough) showed no significant differences as to the period of hospitalization or course of the disease, when compared with the group of patients who did not undergo physiotherapy intervention [13, 14].

Also, in this context, studies conducted in order to prove the efficiency of the thoracic block in reversing atelectasis have only proved that the technique performance did not present significant differences between treated groups and control groups; on the other hand they observed that in the treated group the following can occur: respiratory rates increase, tidal volume reduction, minute volume preservation, and increased number of collapsed alveoli when compared with the control group [11, 12].

Considering the association of the thoracic block technique with an invasive or noninvasive mechanical ventilation apparatus, known in the literature as ITRA, research has achieved significant results in the reversal of atelectasis and, despite the fact that the control group had presented saturation decrease and transient bradycardia during block, the author considered that the ITRA had no adverse effects [9].

The blocking technique consists of changing the flow [15] and acting on the airway driving, without reaching the lung parenchyma, requiring change in volume to achieve the collapsed alveoli. This effect can be achieved by performing the ITRA due to the association of positive pressure, which produces an increased alveolar pressure, increased functional residual capacity, and consequently recruitment of alveolar units in this region [16, 17], a goal achieved in this study when the atelectasis has reversed without showing desaturation or transient bradycardia.

## 4. Conclusion

The use of the thoracic block technique associated with continuous positive airway pressure was efficient in this patient who had atelectasis as a complication of pneumonia and pleural effusion.

## Figures and Tables

**Table 1:** Average parameters assessed during each physiotherapy session.

Variables	Before	During	After 10 min
Heart rate	122	110	111
Respiratory frequency	30	25	24
O_2_ saturation	96%	97%	97%

## References

[1] Mulholland K. (2003). Global burden of acute respiratory infections in children: implications for interventions. *Pediatric Pulmonology*.

[2] Amorim P. G., Morcillo A. M., Tresoldi A. T., Fraga A. D. M. A., Pereira R. M., Baracat E. C. E. (2012). Factors associated with complications of community-acquired pneumonia in preschool children. *Jornal Brasileiro de Pneumologia*.

[3] Williams B. G., Gouws E., Boschi-Pinto C., Bryce J., Dye C. (2002). Estimates of world-wide distribution of child deaths from acute respiratory infections. *The Lancet Infectious Diseases*.

[4] Rodrigues F. E., Tatto R. B., Vauchinski L. (2011). Pneumonia mortality in Brazilian children aged 4 years and younger. *Jornal de Pediatria*.

[5] Riccetto A. G. L., Zambom M. P., Pereira I. C. M. R., Morcillo A. M. (2003). Influence of social-economical and nutritional factors on the evolution to complications in children hospitalized with pneumonia. *Revista da Associação Médica Brasileira*.

[6] Soares P., Barreira J., Pissara S., Nunes T., Azevedo I., Vaz L. (2009). Pediatric parapneumonic pleural effusions: experience in a university central hospital. *Revista Portuguesa de Pneumologia*.

[7] Marchi E., Lundgren F., Mussi R. (2006). Parapneumonic effusion and empyema. *Jornal Brasileiro de Pneumologia*.

[8] Jonhston C., Carvalho W. B. (2008). Atelectasias em pediatria: mecanismos, diagnóstico e tratamento. *Revista da Associação Médica Brasileira*.

[9] Herry S. (2007). Technique Insufflatoire de Levée d’Atélectasie (TILA) en réanimation néonatale. *Kinésithérapie, La Revue*.

[10] Pasquina P., Merlani P., Granier J. M., Ricou B. (2004). Continuous positive airway pressure versus noninvasive pressure support ventilation to treat atelectasis after cardiac surgery. *Anesthesia and Analgesia*.

[11] Sixel B. S., Lemes D. A., Pereira K. A., Guimarães F. S. (2007). Compressão manual torácica em pacientes com insuficiência respiratória aguda. *Fisioterapia Brasil*.

[12] Lima J. G. M., Reis L. F. F., Moura F. M., Souza C. P. V., Walchan E. M., Bergmann A. (2008). Compressão manual torácica em um modelo experimental de atelectasia em ratos wistar. *Fisioterapia em Movimento*.

[13] Perrotta C., Ortiz Z., Roque M. (2005). Chest physiotherapy for acute bronchiolitis in paediatric patients between 0 and 24 months old. *Cochrane Database of Systematic Reviews*.

[14] Pupin M. K., Riccetto A. G., Ribeiro J. D., Baracat E. C. (2009). Comparação dos efeitos de duas técnicas fisioterapêuticas respiratórias em parâmetros cardiorrespiratórios de lactentes com bronquiolite viral aguda. *Jornal Brasileiro de Pneumologia*.

[15] Ribeiro I. F., Melo A. P. L., Davidson J. (2008). Fisioterapia em recém-nascidos com persistência do canal arterial e complicações pulmonares. *Revista Paulista de Pediatria*.

[16] Bohé L., Ferrero M. E., Cuestas E., Polliotto L., Genoff M. (2004). Indications of conventional chest physiotherapy in acute bronchiolitis. *Medicina*.

[17] de Oliveira J. S., Campos T. F., de Oliveira Borja R. (2012). Análise do índice de percepção de esforço na avaliação das pressões respiratórias máximas em crianças e adolescentes. *Revista Brasileira de Crescimento e Desenvolvimento Humano*.

